# Household dairy production and child growth: Evidence from Bangladesh

**DOI:** 10.1016/j.ehb.2018.07.001

**Published:** 2018-09

**Authors:** Samira Choudhury, Derek D. Headey

**Affiliations:** a SOAS, University of London, United Kingdom, United States; b Poverty, Health & Nutrition Division, The International Food Policy Research Institute (IFPRI), United States

**Keywords:** Livestock, Dairy production, Animal-sourced foods, Stunting

## Abstract

Research from richer countries finds that dairy consumption has strong positive associations with linear growth in children, but surprisingly little evidence exists for developing countries where diets are far less diversified. One exception is a recent economics literature using the notion of incomplete markets to estimate the impacts of cattle ownership on children’s milk consumption and growth outcomes in Eastern Africa. In addition to external validity concerns, an obvious internal validity concern is that dairy producers may systematically differ from non-dairy households, particularly in terms of latent wealth or nutritional knowledge. We re-examine these concerns by applying a novel double difference model to data from rural Bangladesh, a country with relatively low levels of milk consumption and high rates of stunting. We exploit the fact that a cow’s lactation cycles provide an exogenous source of variation in household milk supply, which allows us to distinguish between a control group of households that do not own cows, a treatment group that own cows that have produced milk, and a placebo group of cow-owning households that have not produced milk in the past 12 months. We find that household dairy production increases height-for-age Z scores by 0.52 standard deviations in the critical 6–23 month growth window, though in the first year of life we find that household dairy supply is associated with a 21.7 point decline in the rate of breastfeeding. The results therefore suggest that increasing access to dairy products can be extremely beneficial to children’s nutrition, but may need to be accompanied by efforts to improve nutritional knowledge and appropriate breastfeeding practices.

## Highlights

• Milk production/consumption strongly linked to child growth in European and African populations.
• This is the first study to look at dairy production and child growth in an Asian setting.
• Uses unique survey data to exploit quasi-experimental variation in exposure to milk production.
• Milk production increases child HAZ scores by 0.52 standard deviations in the 6–23 month age range.
• However, milk production is associated with a 20-point decline in exclusive breastfeeding.

## 1 Introduction

Worldwide, child undernutrition is increasingly recognized as a significant global health problem and a major constraint to economic development. Child undernutrition is associated with almost 3.1 million child deaths (Black et al., 2013), impaired cognitive development in early childhood (Walker et al., 2011; Grantham-McGregor et al., 2007), reduced school attainment in childhood, and lower labour productivity and wages in adulthood (Shekar et al., 2006; Victora et al., 2008; Hoddinott et al., 2008). Nutritionists, moreover, have increasingly emphasized that it is good nutrition in *early* childhood – in utero and the first 24 months after birth – that is truly critical for ensuring healthy growth (Shrimpton et al., 2001; Victora et al., 2010).

A particularly striking nutritional feature of developing country populations is that growth faltering appears to be particularly pronounced from roughly 6 months of age to 20 months of age, a period that coincides with the introduction of complementary foods that are often low in high quality protein and micronutrients, such as rice, wheat, maize or starchy roots and tubers. Previous research has found that calorie intake alone is not always a strong predictor of child growth in developing countries settings (Griffen, 2016), perhaps because calorie requirements for infants are relatively modest. Instead, many researchers point to low consumption of animal-sourced foods (ASFs) as a critical constraint (Allen, 2003; Brown, 2003; Demment et al., 2003; Headey and Hoddinott, 2016; Neumann et al., 2002; Puentes et al., 2016; Randolph et al., 2007). Indeed, in the absence of fortified foods, young children cannot meet their micronutrient needs without daily intake of ASFs (PAHO/WHO, 2003).

Dairy constitutes a particularly important complementary ASF for young children because of the familiarity of its taste to exclusively breastfed children, and because of its nutritional profile. Dairy is high in all three macronutrients (energy, fat and protein), as well as important micronutrients such as vitamin A, vitamin B12, and calcium (Murphy and Allen, 2003). Moreover, like other ASFs, dairy contains several essential fatty acids that are hypothesized to be critical for processes of cellular growth and bone formation (Semba et al., 2016). Dairy has a protein digestibility corrected amino acid score (PDCAAS) of about 120%. Many studies also suggest dairy intake affects child growth through a stimulating effect on plasma insulin-like growth factor 1 (IGF-1). Milk also contains minerals such as potassium, magnesium, and phosphorus, that could also be a factor stimulating growth, as well as lactose.

Consistent with this biological evidence, a range of research has linked linear growth to childhood dairy consumption, albeit mostly in developed country samples (Iannotti et al., 2013; de Beer, 2012; Dror and Allen, 2011; Wiley, 2005, 2009; Sadler & Catley 2009). In developing countries there have been remarkably few efficacy trials of dairy supplementation on growth in infants or young children, though several dairy consumption programs have demonstrated some impact on linear growth at older ages (Iannotti et al., 2013).^1^

Because of the limitations of experimental evidence on this subject, economists have increasingly utilized observational or quasi-experimental analyses to explore the associations between dairy production and child nutrition outcomes in less developed settings. In economic history studies, Baten (2009, 2014) tests a “protein proximity” hypothesis with 19^th^ Century European military recruitment data from Central Europe. Utilizing the idea that fresh milk in these economies could not be traded over large distances, he finds that adult men in closer proximity to dairy production were substantially less likely to be too short for military recruitment. Still other studies hypothesize that trends in milk consumption explain longer term secular improvements in heights at later stages of economic development, such as 20^th^ Century Japan (Takahashi, 1984) and India (Mamidi et al., 2011). A recent paper also examined adult heights in 42 European countries with varying levels of development. Even after controlling for genetic factors, they found that the national supply of protein from dairy products was the single strongest predictor of adult stature (Grasgruber et al., 2014). A related study of 105 countries from different continents also found strong associations between average milk consumption levels and adult male heights (Grasgruber et al., 2016).

In contemporary developing countries several studies have examined associations between household livestock ownership and child growth outcomes, though not all studies focus on milk-producing animals specifically. Like Baten (2009, 2014) these studies assume (often implicitly) that fresh milk is generally non-tradable and not a perfect substitute for powdered milk. Hoddinott et al. (2015) use two large surveys from Ethiopia to specifically explore the association between cattle ownership, dairy consumption and HAZ scores. They cite the fact that 90% of milk produced in rural Ethiopia is consumed by the household producing it, implying that cattle ownership ought to be a very strong predictor of regular dairy intake. Consistent with that conjecture they find strong positive associations between cattle ownership and HAZ (as high as 0.47 standard deviations in the 12–23 month age-range). They also implement placebo tests to explore the concern that cattle ownership proxies for generic wealth effects on child nutrition.

Rawlins et al. (2014) evaluate Heifer International’s dairy cow and goat ownership programs in Rwanda, albeit in a non-randomized quasi-experimental design with a small sample of 217 children aged 0–59 months (precluding the possibility of detailed age disaggregation). They find that children from households who received a goat 12 months prior to the time of the survey saw no growth differential over controls, whereas transfers of pregnant cows (high-productivity foreign breeds) improved height-for-age Z scores by 0.57 standard deviations, a large but imprecisely estimated effect. Similarly, Kabunga et al. (2017) use matching methods to gauge the impacts of adoption of improved dairy cow varieties on HAZ of children aged 6–59 months. They find HAZ impacts of 0.48-0.49 standard deviations, though also some evidence of larger impacts for household with greater herd sizes or larger acreage.^2^

Overall, there is fairly consistent evidence that dairy cow ownership is associated with child growth in poorer populations, although there are several limitations and caveats surrounding this evidence. First, the evidence is confined to East African localities where cattle ownership is relatively common, so external validity is a concern. Second, this literature potentially suffers from several internal validity issues, including the confounding role of livestock as a source of imperfectly measured rural wealth, and potential concerns over associations between livestock ownership and ethnicity.^3^ Another outstanding concern not addressed in the previous literature is that the availability of cow’s milk leads to premature cessation of breastfeeding by mothers. Exclusive breastfeeding is strongly recommended for the first 6 months of life, especially in developing country settings, because of its critical role in preventing diarrhea and respiratory infections (Horta and Victora, 2013), and because cow’s milk can stress a newborn’s immature kidneys and irritate the lining of the stomach and small intestine, leading to blood loss and iron-deficiency anemia (FAO, 2013).

In light of these limitations, this paper utilizes a unique dataset to attempt a more comprehensive assessment of the nutritional implications of dairy production and consumption in Bangladesh. Bangladesh is a particularly important case study in the context of dairy production. In addition to its high rates of stunting (36%), Headey and Hoddinott (2016) emphasize that Bangladesh has an under-diversified food supply, with FAO data suggesting that ASFs account for less than 5% of total calories supplied (Headey and Hoddinott, 2016). This situation partly stems from exceptionally low levels of milk consumption, which in per capita terms is less than half that of neighbouring India (Headey and Hoddinott, 2016). A likely explanation of this is the country’s exceptionally severe land constraints (and hence feed constraints), with average farm sizes in Bangladesh averaging just half a hectare, and rural landlessness widespread. It may also be that cultural norms – historical unavailability of milk – has kept demand for milk relatively low.

In this paper we use the nationally representative Bangladesh Integrated Household Survey (BIHS) of rural areas, which was conducted over two rounds in 2011/2012 and 2015. Uniquely for such a large survey, this dataset contains rich information both on nutrition outcomes, individual food consumption, agricultural assets and production, and a range of other potential determinants of nutrition. Methodologically, we propose a novel difference-in-difference approach to assessing the impact of dairy cow ownership on child nutrition outcomes, by distinguishing between households with lactating dairy cows that have produced milk over the past 12 months (*treatment*), households with cows that have not produced milk in the past 12 months (*placebo*), and households that do not own any dairy cows (*control*). We note that this is not a placebo in the medical definition (according to which a person consumes a treatment of no intended therapeutic value), but in the sense that non-lactating cows might have a similar long run economic value any direct milk supply to the household. This distinction between the *treatment* and *placebo* emerges from the fact that smallholder dairy producers in Bangladesh typically only own a few cows because of the extreme land and feed constraints mentioned above. Specifically, 80% of Bangladeshi farmers in our nationally representative sample own just 1–2 cows and no farmers in our sample own more than 4 animals. Given that at any given time all or some of these cows will not be lactating – since there is a minimum 12-month inter-calving cycle for each animal even among the most technologically sophisticated dairy producers – there is a non-trivial proportion of dairy cow owners in Bangladesh who would be unable to produce milk on a continuous basis for exogenous biological reasons.^4^

In effect, then, the combination of small herds and a biologically determined component of the lactation cycle potentially creates a valid placebo group of children who are treated with cows that have not produced any milk. We therefore test three hypotheses:

(i) Children in *treatment* group will be taller than children in *control*;
(ii) Children in the *placebo* group will not be taller than the *control*; and
(iii) Children in *treatment* group will be taller than *placebo* group children.

In addition to these tests we also examine whether livestock ownership or milk production is associated with other observable potentially confounding factors, such as maternal nutritional knowledge and empowerment, and overall child diversity, exclusive of milk. And unlike previous studies in this literature we explore the policy-relevant question of whether access to a stable household level supply of dairy products leads to substitution between breastfeeding and dairy milk intake.

We find that milk production is strongly associated with linear growth, but only for children in the crucial first 1000 days of life (particularly the 12–23 month range). The effects we observe are very close in magnitude to those observed in the aforementioned quasi-experimental study by Rawlins et al. (2014) for Rwanda and Kabunga et al. (2017) for Uganda, but larger than the more observational study by Hoddinott et al. (2015) who analyse the impacts of owning any cow, rather than milk-producing cows specifically (rendering their results more like an intent-to-treat analysis). Null results for the *placebo* group also lend credence to the identification assumptions underlying our approach, as do additional placebo tests which rule out systematic differences in nutritional knowledge and women’s empowerment. However, we do find some evidence of potentially harmful effects of household dairy availability on breastfeeding in the first year of life, suggesting dairy-oriented nutrition strategies need to proactively promote exclusive breastfeeding in the first six months to prevent premature substitution into dairy.

The remainder of this paper is organized as follows. Section 2 describes the data and the methods used to analyse them. Section 3 tests associations between different ASF production and various nutrition outcomes. Section 4 provides some important sensitivity tests and extensions, and Section 5 concludes with a discussion of the implications of these findings for programs and policies, as well as for future research.

## 2 Conceptual model, data and methods

As outlined above, our objective in this paper is to test for significant differences in milk consumption and child growth between household groups that are defined by dairy production and cow ownership. Previous papers in this literature have tended to focus on a comparison between a “treatment group” of households that own any dairy cow and a “control group” of households that do not own any dairy cows. In our data we instead narrow the definition of treatment households to those that owned cows that actually produced milk in the past 12 months (hereafter *treatment*). We then define what can be thought of as a “placebo group” of children exposed to cows that had not produced any milk in the past 12 months (note that we think of this group as a placebo because the treatment is not milk per se - in which case the placebo would be a milk substitute - but milk-producing cows). In an ideal experimental design children would be randomly assigned across groups, but in observational settings a significant concern is that there may be systematic nutrition-relevant differences between treated and non-treated children (*e.g.* wealth, nutritional knowledge, women’s empowerment). Achieving more experimental conditions might therefore require extensive control for potential confounding factors.

The conceptual model described in Hoddinott et al. (2015) is a useful starting point for thinking about the various factors that might influence household decisionmaking processes with respect to dairy production, dairy consumption and child nutrition. They posit a household utility model in which child nutrition is one argument. Nutritional status is itself a function of nutrient (food) intake, as well as nutritional knowledge, culture, healthcare, genetic endowments, and locational characteristics (such as the prevalence of disease; access to information about good child care practices). In a world of perfectly functioning markets, nutrient intake would be primarily influenced by income, and households could sequentially maximize farm and nonfarm income before deciding how to spend that income so as to maximize nutrition outcomes subject to other arguments in the utility function. However, the perishability of milk in poorly developed value chains renders household production and consumption decisions non-separable. In other words, if households struggle to access affordable milk *via* markets, they could opt to own dairy cows. This implies that the decisions to own dairy cows and/or produce milk may be endogenous, influenced as it is by nutrition knowledge and farm production parameters such as the availability of capital (income, savings, wealth), access to land (feed), access to input and output markets to obtain feed and sell produce, household labour supply, farm management skills, and the role of women in household decisionmaking, including dairy production and feeding practices.

Since omission of these kinds of factors could lead to biased coefficients on the impacts of cattle ownership or milk production on child growth, our empirical models need to control for these factors as extensively as possible. Fortunately, the Bangladesh Integrated Household Survey (BIHS) not only contains detailed data on children’s food intake and nutrition outcomes, but also an exceptionally rich array of data on income, wealth, agricultural production and assets, access to markets, women’s empowerment and women’s nutrition knowledge (IFPRI, 2016). BIHS is also a large survey representative of rural Bangladesh that has been implemented in two rounds (2011/2012 and 2015) and constitutes a panel for the majority of households. However, because we are interested in child growth in the first 5 years of life – particularly the 12–23 month period – we treat both rounds as repeated cross-sections rather than a panel.^5^ The combined rounds make up a sample of 11,796 households (some surveyed twice), which includes 4268 pre-school children aged 0–59 months.

Height-for-age Z scores (HAZ), using the World Health Organization’s global child growth reference standards (WHO, 2006), constitutes our primary outcome of interest. As noted above, from 6 months to around 24 months growth faltering tends to be particularly pronounced in developing country populations due to prolonged nutritional deficiencies associated with inappropriate complementary feeding and repeated or chronic infections (Victora et al., 2010). It is also common to define children as stunted if HAZ falls below -2, though statistical epidemiologists have strongly argued against using dichotomous dependent variables, as it unnecessarily discards valuable information and reduces precision (Royston et al., 2006). However, we report stunting results as an extension to our main HAZ results.

In this paper our interest is in dairy production-dairy consumption pathways, rather than dairy production-income/wealth pathways (in principle, income from any source could improve diets). Our regression models therefore control for household expenditure and wealth, but our dataset also allows us to examine whether dairy consumption is likely to be the main mechanism linking cow ownership to child growth by using additional data on children’s consumption of various foods as well as household data on how different foods were obtained. In terms of the former we primarily focus on children’s consumption of dairy products in the past 24 h, defined as a dichotomous indicator. To help rule more generic income-based pathways we also use a dietary diversity score (0–6 food groups) that excludes dairy, as well as estimates of children’s total calorie consumption (excluding breastmilk). Our expectation is that dairy production influences dairy consumption, but not non-dairy dietary diversification or total calorie intake. We can also explore how households sourced different foods since the BIHS asks respondents to estimate the proportion of each food provided through market purchases, provided by other sources, or provided by home production. We also note that, in principle, these consumption data might also be used to examine the impacts of dairy consumption on child growth. However, a critically important limitation of consumption data is that they are based on short recall periods (24-hour or weekly recall), meaning that they are potentially quite poor indicators of regular consumption of milk in the past 12 months or more (Thorne-Lyman et al., 2014).

This measurement problem with short-recall consumption suggests that longer recall questions on milk production may be a much better indicator of regular access to dairy products in settings where markets for perishable products are highly imperfect. However, since long-recall production quantity indicators also suffer from bias we use a simpler dichotomous indicator of whether or not milk was produced in the last 12 months – along with cow ownership - to define our *treatment*, *placebo* and *control* groups.

Clearly these groups are not the result of random assignment, although we can use multivariate regressions to reduce the biases of confounding factors that influence cow ownership or lactation decisions. We first assess the determinants of milk production, with the expectation that cattle herd size (female and males) is a key observable driver that we can subsequently control for in our main HAZ regressions. We then use multivariate reduced form regressions to control for a broader range of potential confounding factors. In addition to dairy herd size, we were also concerned that cattle ownership may simply reflect more generic livestock wealth, so we extensively control for other forms of livestock (bullock/buffalo, goat, sheep, chicken, duck and other birds) and aggregate livestock into an index of Tropical Livestock Units (TLU), which can be thought of as a measure of aggregate livestock wealth. The remaining control variables are more common to most nutrition specifications, and to estimation of health production functions, such as Todd and Wolpin (2007) and Hoddinott et al. (2015). This includes child characteristics (sex, age, breastfeeding status), parental characteristics (age and schooling), household characteristics (per capita monthly expenditure, the aggregate value of 26 household assets, hectares of cultivable land owned, household toilet and water access, access to electricity, exposure to NGO services) and several community characteristics (distances to the nearest weekly/periodic outdoor market, and to the nearest town and to the nearest health centre). Our regressions also include fixed effects for all 65 districts in which the BIHS was conducted. Clearly the main coefficient of interest is that pertaining to the *treatment* group, which we interpret as the effect of dairy availability on child growth net of any impacts of dairy production on other inputs into the health production function, such as income, or changes in breastfeeding. However, we also test for significant differences between the coefficients for *treatment* and *placebo*, and whether the coefficient for *placebo* is significantly different from zero (*i.e.* from the *control*, the omitted control group). A significant coefficient on *placebo* would suggest that cattle ownership influence HAZ through channels other than dairy consumption.

A biological issue of paramount importance is the need to explore age-specific variation in the sensitivity of children’s growth to exposure to dairy production, an issue emphasized in Hoddinott et al. (2015). For the HAZ analysis we primarily focus on children 6–23 months and 24–59 months, as well as smaller age intervals. The biology of growth identified in Victora et al. (2010) suggests that most growth faltering takes place in the 6–23 month window, so dairy consumption in this period ought to be critical. We do report results for older children (24–59), although it is not clear that our 12-month dairy production indicator should predict stronger growth because of misclassification errors. That is, some 24–59 month children who may have consumed dairy in the past 12 months (according to our indicator) may not have consumed dairy in their critical 6–23 month window.

In our extensions to the basic model we also examine two indicators that were not collected for all households and would therefore entail sample restrictions: maternal nutrition knowledge score and a maternal empowerment score based on women’s control over and ownership of various agricultural assets. We use these indicators as dependent variables to test whether dairy producing households are significantly more likely to have mothers with better nutrition knowledge or greater empowerment. Here we test the null hypotheses that the coefficient on *treatment* is equal to that of *placebo* and *control*. Rejection of this null would cast doubt might suggest that part of the estimated effects of milk production on HAZ pertains to greater nutrition knowledge or empowerment. We also estimate alternative HAZ specification where production quantities of milk are used in place of the dummy variable for any milk produced. This is not our preferred indicator because of concerns over measurement error, related to the challenges of accurately recalling production over a long period, but we nevertheless consider it a useful alternative test.

## 3 Main results

### 3.1 Descriptive statistics

**Fig. 1:**
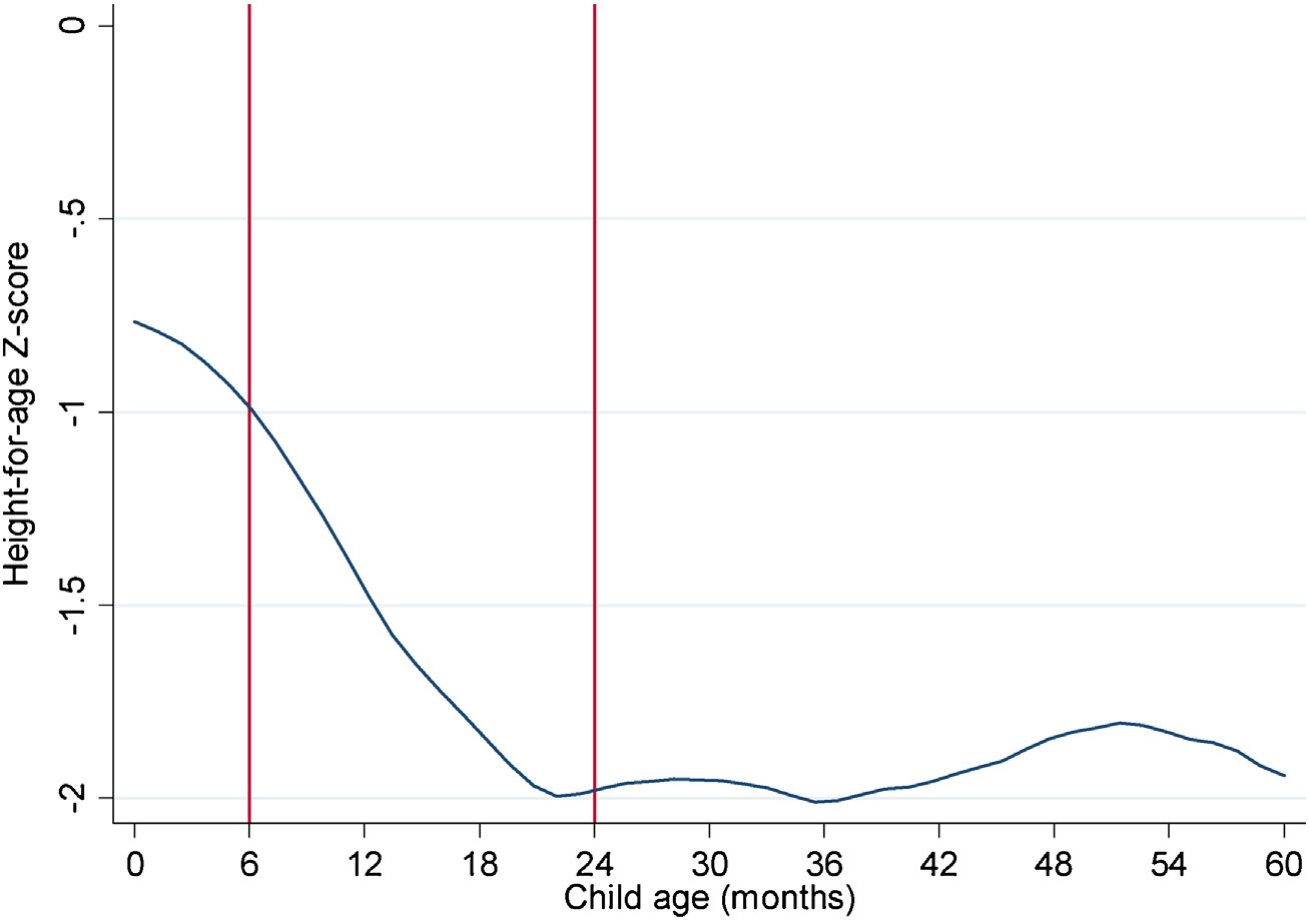
A local polynomial graph of height-for-age Z scores by child age in rural Bangladesh.

Source: Authors’ estimates from Bangladesh Integrated Household Survey 2011, 2015

Table 1 provides descriptive statistics for the key variables for a sample of children 0–23 months of age. Fig. 1 also reports a local polynomial smoother curve (LPOLY) of HAZ scores against child age to reveal the dynamics of growth faltering in rural Bangladesh. There are several broad inferences to be made from these results.

**Table 1:** Descriptive statistics for child, household and community level data for a sample of children 0–23 months of age.

Variable	Obs	Mean	Std. Dev.	Min	Max
Height-for-age Z score (HAZ)	1596	−1.37	1.59	−5.87	5.83
Stunted	1,596	0.34	0.47	0.00	1.00
*Treatment*: Owns cow(s), produced milk	1,596	0.14	0.34	0.00	1.00
*Placebo*: Owns cow(s), no milk produced	1,596	0.08	0.27	0.00	1.00
Child consumed dairy last 24 hrs	1,588	0.22	0.41	0.00	1.00
Quantity milk produced (liters), last 12m	1,312	43.74	140.42	0.00	1500.00
Number of bullocks	1,596	0.43	0.91	0.00	8.00
Number of cows	1,596	0.38	0.83	0.00	4.00
Owns cow, produced milk	1,596	0.14	0.34	0.00	1.00
Owns cow, no milk	1,596	0.08	0.27	0.00	1.00
Owns goat/sheep	1,596	0.15	0.35	0.00	1.00
Owns poultry/duck/other birds	1,596	0.63	0.48	0.00	1.00
Owns/produces fish	1,596	0.29	0.45	0.00	1.00
Total livestock owned (TLUs)	1,596	0.68	1.20	0.00	25.80
Currently breastfed	1,588	0.50	0.50	0.00	1.00
Log per capita monthly expenditure	1,596	7.69	0.52	6.37	10.71
Log value of household assets	1,596	10.99	1.26	6.17	17.96
Land area cultivated	1,596	0.22	0.43	0.00	6.43
Access to electricity	1,596	0.54	0.50	0.00	1.00
Mother primary education	1,596	0.54	0.50	0.00	1.00
Mother secondary education	1,596	0.06	0.24	0.00	1.00
Mother tertiary education	1,596	0.04	0.19	0.00	1.00
Household head primary education	1,596	0.34	0.47	0.00	1.00
Household head secondary education	1,596	0.08	0.28	0.00	1.00
Household head tertiary education	1,596	0.04	0.19	0.00	1.00
Access to water supply	1,596	0.78	0.41	0.00	1.00
Access to improved toilet	1,596	0.32	0.47	0.00	1.00
Distance to regular bazaar (km)	1,596	1.79	1.79	0.00	25.00
Distance to health centre (km)	1,596	6.10	6.34	0.00	70.00
Loan from NGO	1,596	0.46	0.50	0.00	1.00
Male child	1,596	0.52	0.50	0.00	1.00
Household size	1,596	5.55	2.29	2.00	21.00
Maternal age	1,596	25.84	5.67	16.00	51.00
Nutritional knowledge score	1,596	8.79	1.91	0.00	14.00
Maternal empowerment score	1,113	0.70	0.23	0.10	1.00
Child diet diversity (6 groups, excluding dairy)	1,588	1.85	1.76	0.00	6.00
Child Calorie Intake (kcal)	1,596	286.25	331.50	0.00	2919.28

First, the sample of children is highly undernourished, consistent with other nationally representative surveys of Bangladesh. Mean HAZ scores are −1.37, and one third of children are stunted (by age two fully half are stunted). However, consistent with previous research (Victora et al., 2010), most of the growth faltering in Bangladesh occurs in the 6–23 month window, as shown by the red vertical lines in Fig. 1. This accelerated period of growth faltering could partially be due to poor diets. Notably, the percentage of all children who consumed dairy in the past 24 h is just 22%, which is particularly low given that in more developed societies many children would consume milk on a daily basis. Consistent with low milk consumption is the low ownership of milk producing cows (14%), while a further 8% own a cow that has not produced milk in the past 12 months.

### 3.2 Determinants of milk production

The higher socioeconomic status of *treatment* might imply that any apparent benefits of dairy production partially reflect the benefits of greater socioeconomic status. This points to the importance of multivariate regression models saturated with a wide array of controls, as well as the importance of placebo tests. However, we can also examine the determinants of milk production across among households that own at least one cow (*treatment* and *placebo*) to assess the relative importance of herd size *versus* other socioeconomic indicators. On biological grounds one would expect milk production to be strongly associated with herd size, including the number of both female animals and male animals. Owning more female animals obviously reduces the risk that the herd as a whole will not have produced any milk in the past 12 months. However, without male animals, producers would need to either rent in bulls, or access artificial insemination services. While the latter are common in Bangladesh, previous research points to poor farm management practices reducing the success of artificial insemination services (see footnote 3).

Table 2 reports the results for those variables that statistically explain whether or not a cow-owning household has produced milk in the past 12 months. With the exception of maternal age, the only significant predictors of dairy production status are indicators of herd size; coefficients on the range of other indicators of household socioeconomic status are all insignificant, individually and jointly. The results suggest that milk production status is non-linearly related to herd size: owning 2 dairy cows or 1 bullock greatly increases the probability of producing milk in the past year, but additional animals do not much alter these probabilities.

**Table 2:** Statistically significant determinants of milk production (treatment status) in past 12 months among households that owned at least one cow (linear probability model).

	(1)
	Produced milk in past 12 months (i.e. treatment group status)
Owns 2 cows	0.349^***^
	(0.052)
Owns 3 cows	0.460^***^
	(0.054)
Owns 4 cows	0.334^***^
	(0.083)
Owns 1 bullock	0.267^***^
	(0.054)
Owns 2 bullocks	0.363^***^
	(0.049)
Owns 3 bullocks	0.268^***^
	(0.081)
Owns 4 bullocks	0.173*
	(0.100)
Owns 5 bullocks	0.853^***^
	(0.071)
Owns 6 bullocks	0.162*
	(0.091)
Mother’s age	0.008^***^
	(0.003)
All livestock ownership variables?	Yes
Controls for age and gender?	Yes
Other socioeconomic controls?	Yes
District fixed effects?	Yes
Observations	728
R-squared	0.405

Notes: These are linear probability estimates, with standard errors are in parentheses, clustered at village level.

^***^p < 0.01, **p < 0.05, *p < 0.1.

Control variables are described in Table 1.

**Fig. 2:**
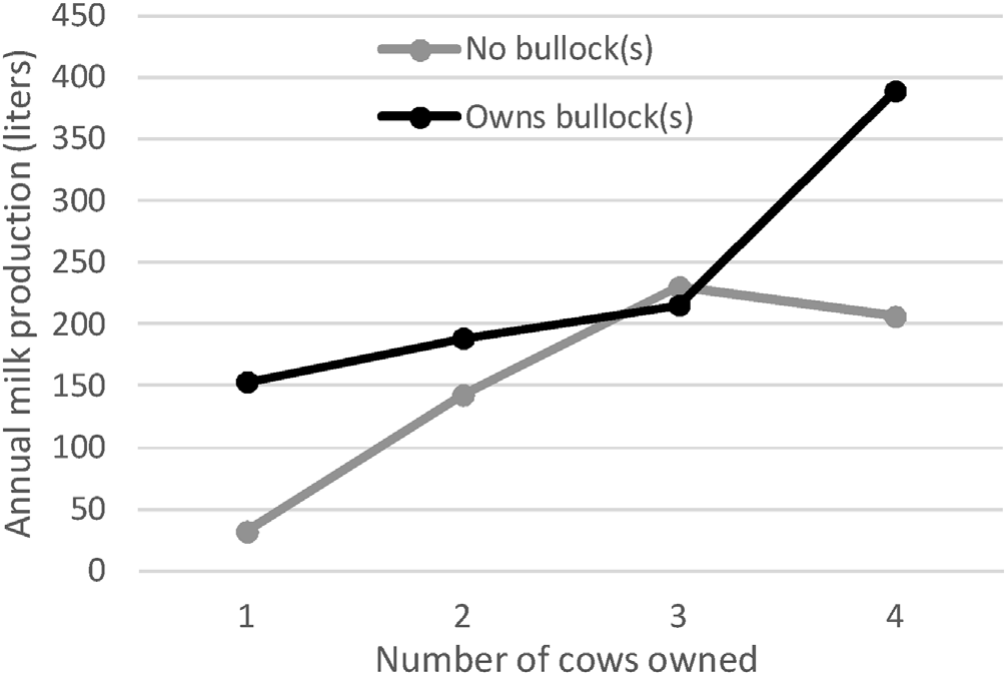
Mean milk production as a function of the number of cows owned, for households own and do not own bullocks.

Source: Authors’ estimates from Bangladesh Integrated Household Survey 2011, 2015

Fig. 2 explores the relationship between herd size and annual milk production on the y-axis and the number of cows owned on the x-axis. However, we plot a curve for households that own at least one bullock, as well as those that do not, in order to examine interaction effects. The results reveal the expected finding that owning just one cow with no bullock results in very low levels of milk production because there is a high likelihood that this single cow may not have been lactating at any time in the past 12 months. Owning more cows greatly improves milk production. Moreover, the returns to owning one cow and at least one bullock are fairly high, and not greatly increased by owning more cows.

### 3.3 Associations between dairy production and dietary indicators

**Fig. 3:**
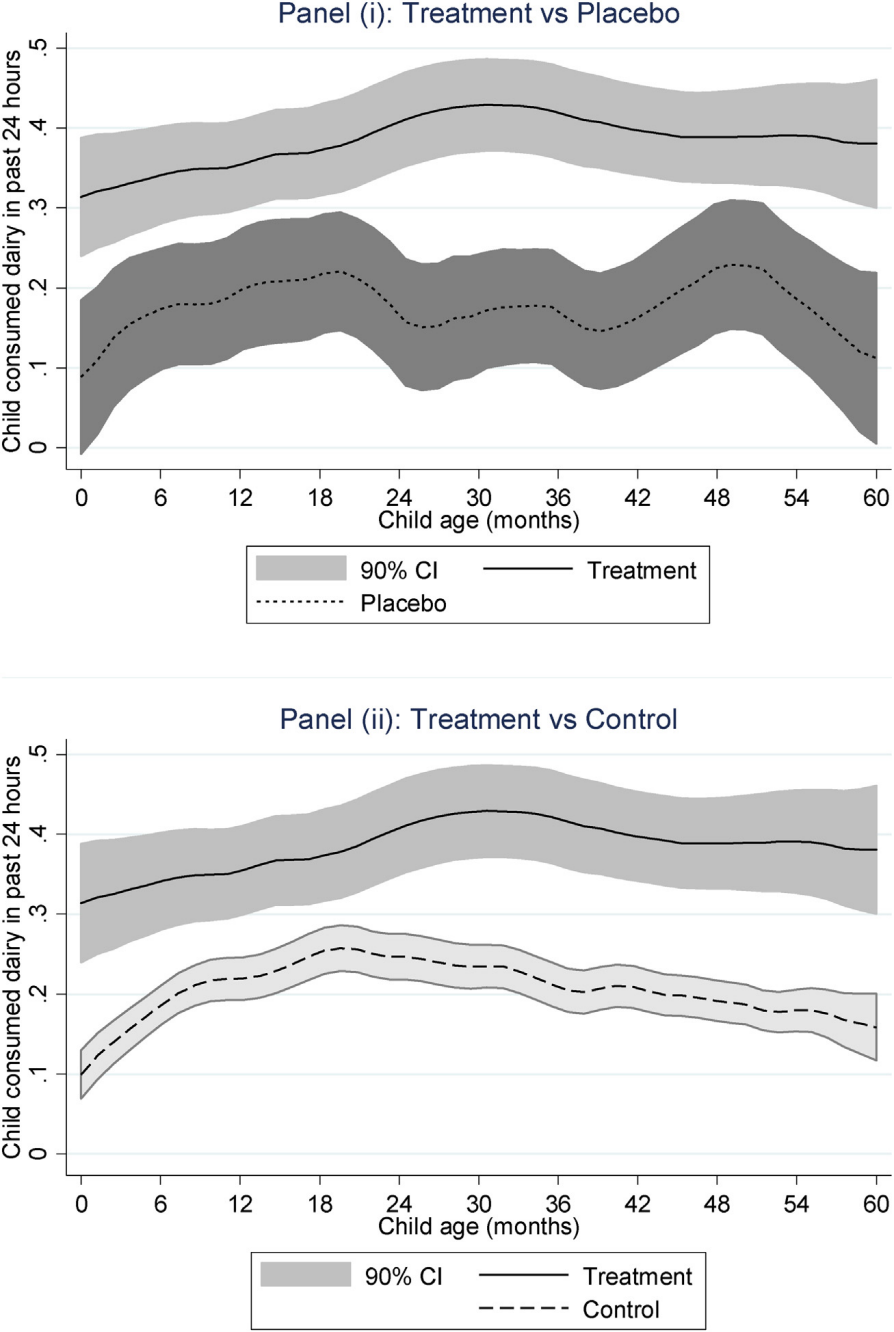
Local polynomial smoothing estimates of dairy consumption against child age by treatment group, with 90% confidence intervals.

Source: Authors’ estimates from Bangladesh Integrated Household Survey 2011, 2015

Fig. 3 shows local polynomial smoother plots of the relationship between 24-hr dairy consumption and child age, with 90% confidence intervals (CIs). We use 90% CIs in order to implement a one sided test at the 5% level that *treatment* status is associated with higher HAZ. Panel (i) compares *treatment* to *placebo*, while Panel (ii) compares *treatment* to *control*. The 90% CIs do not overlap in either panel, indicating that *treatment* children have significantly higher levels of dairy consumption compared to the *placebo* or *control* groups throughout the 0–59 month age range. The magnitude of the difference between *treatment* and *placebo* and *control* varies between 15–25 percentage points depending on the age of the child.

Table 3 examines this relationship in a multivariate regression model with a full set of controls, but also looks at whether milk production has any impact on non-dairy dietary diversity and child calorie intake. Results in Regression (1) suggest that milk production leads to approximately a 14-point increase in milk consumption, although some of the difference in milk consumption across groups observed in Fig. 3 is likely driven by differences in socioeconomic status (household expenditure, maternal education) across groups.^6^ Another striking result from Fig. 3 is that many children under the age of 12 months consume cow’s milk, even though recommendations (albeit based more on developed country samples) recommend milk consumption be initiated only at 12 months (FAO, 2013). Moreover, previous research using the same dataset suggests that children are often given the lion’s share of a household’s milk supply in Bangladesh (Sununtnasuk and Fiedler, 2017).

**Table 3:** Associations between livestock ownership and dietary indicators among children 6–59 months (linear probability and least squares regressions).

	(1)	(2)	(3)
	Consumed dairy, last 24 hrs	Dietary diversity score (0-6), excluding milk	Total calorie intake (kcal)
*Treatment group (vs control)*	0.139***	−0.044	15.300
	(0.042)	(0.094)	(25.733)
*Placebo group (vs control)*	−0.019	0.022	88.038***
	(0.036)	(0.084)	(27.536)
Owns buffalo/bullock	−0.015	−0.018	28.835
	(0.022)	(0.066)	(19.502)
Owns goat/sheep	−0.021	−0.071	−0.896
	(0.021)	(0.057)	(22.060)
Owns poultry/duck/other birds	0.015	0.036	−4.515
	(0.015)	(0.047)	(18.027)
Owns/produces fish	−0.022	0.041	6.810
	(0.019)	(0.047)	(22.133)
Total Livestock Units (TLU)	0.004	0.025	−11.805
	(0.015)	(0.029)	(7.631)
All controls	Yes	Yes	Yes
District fixed effects?	Yes	Yes	Yes
Observations	3,352	3,352	3,362
R-squared	0.172	0.362	0.495
Wald tests (p-values):			
β(*Treatment*) > β(*Control*)	0.001***	0.970	0.98

Notes: These are least squares or linear probability estimates, with standard errors are in parentheses, clustered at the village level. ***p < 0.01, **p < 0.05, *p < 0.1.

Finally, Table 3 also examines whether there are systematic differences in non-dairy dietary diversity across groups, as well as total calorie intake (exclusive of breastmilk). We find no significant associations between *treatment* and these two dietary indicators, although the *placebo* group has higher calorie intake than the *treatment* or *control* groups. The lack of any impact on non-dairy dietary diversity suggests the results may not be confounded by socioeconomic differences between groups (Hoddinott, Headey and Dereje 2014). The lack of a significant impact on calories suggests that milk consumption is not primarily operating through increasing a child’s overall calorie intake in this context.

### 3.4 Associations between dairy production and child growth

Table 4 presents least squares regression results with a full set of control variables, stratified by 6–23 months, 24–59 months, and then by series of overlapping 12-month age brackets used to further corroborate the importance of milk in this 6–23 month window. The most striking result is the large 0.52 standard deviation (SD) difference between *treatment* and *control* children in the 6–23 month window; a difference which entirely disappears in the 24–59 month window. The latter result is likely explained by the fact that there may be low serial correlation between milk production in the past year and milk production in earlier years, precisely because of variations in lactation cycles among small-scale dairy producers. In columns (3) and (4) we see that the results are consistent across the 6–17 month and 12–23 month windows, though column (4) shows a relatively large but insignificant coefficient on the placebo group coefficient, while column (5) confirms that the benefits of milk production are no longer apparent once we move above the 23 month threshold. We interpret this as evidence that milk consumption has its largest impact in the first 1000 days; as the age range moves beyond ∼23 months the 12 month recall becomes a more imprecise indicator of whether the child actually consumed milk in the 6–23 month period. Further confirmation that the results are strongest in the 6–23 month period is provided by Wald tests of significant differences between the treatment and placebo coefficients in the 6–23 month, 6–17 month and 12–23 month ranges. This suggests that it is milk production, not cattle ownership per se, that yields sizeable benefits for linear growth in early childhood.

**Table 4:** Associations between HAZ and exposure to milk production across different age groups (least squares regressions).

	(1)	(2)	(3)	(4)	(5)	(6)
	6-23 months	24-59 months	6-17 months	12-23 months	18-29 months	24-35 months
*Treatment group (vs control)*	0.520^***^	0.040	0.548**	0.557^***^	0.473^***^	−0.009
	(0.165)	(0.120)	(0.235)	(0.182)	(0.137)	(0.186)
*Placebo group (vs control)*	0.162	0.173	0.028	0.094	0.371	0.106
	(0.162)	(0.116)	(0.226)	(0.257)	(0.247)	(0.239)
All controls variables?	Yes	Yes	Yes	Yes	Yes	Yes
District fixed effects?	Yes	Yes	Yes	Yes	Yes	Yes
Observations	1,154	2,384	869	788	800	830
R-squared	0.194	0.129	0.203	0.181	0.158	0.168
Wald tests (p-values):						
β(*Treatment*) > β(*Control*)	0.05**	0.17	0.02**	0.07*	0.69	0.59

Notes: These are least squares estimates, with standard errors are in parentheses, clustered at village level.

^***^p < 0.01, **p < 0.05, *p < 0.10.

“All controls” includes controls for ownership of other livestock and total TLUs (livestock wealth), as well as the full set of socioeconomic controls described in Table, a gender dummy and monthly dummies for child age, as well as district fixed effects.

### 3.5 Extensions

In addition to the results above we also engaged in a series of extensions designed to explore some additional complexities in the associations examined above. We first tested for differential impacts of *treatment* on boys and girls, but found no statistically significant differences in results for the age ranges above. We also tested for interactions between *treatment* status and maternal empowerment scores and maternal nutritional knowledge on the grounds that these might be mediating factors, but all interactions were insignificant. We also included empowerment scores and knowledge scores as dependent variables to see if these might be potential confounding factors, but *treatment* status had no significant impact on either variable (results available on request).

In Table 5 we used stunting status (HAZ<-2) as the dependent variable. Stunting is a widely used public health measure, although using a dichotomous indicator rather than a continuous indicator effectively discards information and is likely to reduce precision. The pattern of results in Table 5 are very similar to those reported in Table 4, although the Wald tests no longer report statistically significant differences across the treatment and control groups (seemingly due to the expected increase in imprecision). That caveat aside, the results imply that regular dairy consumption has strong impacts on stunting, although *treatment-control* and *treatment-placebo* comparisons yield quite different inferences. Among children 6–23 months the model predicts a 10.4-point reduction in stunting relative to the control group. However, the placebo group also has a large, negative but statistically insignificant coefficient that – interpreted literally – would imply only a 2.4-point reduction in stunting from exposure to treatment. Among children 12–23 and 18–29 months the point estimates on *treatment* are even larger, implying 14 and 22-point reductions in the risk of stunting relative to control, and 8.4-point and 11.3-point reductions relative to the *placebo* group. Overall, then, these results for stunting status are broadly similar to the HAZ results in Table 4, although it is no longer possible to establish statistically significant differences between *treatment* and *placebo*.

**Table 5:** Associations between stunting status and exposure to milk production across different age groups (linear probability model).

	(1)	(2)	(3)	(4)	(5)	(6)
	6-23 months	24-59 months	6-17 months	12-23 months	18-29 months	24-35 months
*Treatment group (vs control)*	−0.104**	−0.049	−0.034	−0.136**	−0.223***	−0.135
	(0.046)	(0.041)	(0.059)	(0.063)	(0.058)	(0.087)
*Placebo group (vs control)*	−0.080	−0.048	−0.028	−0.052	−0.110	−0.091
	(0.058)	(0.047)	(0.085)	(0.085)	(0.075)	(0.079)
All controls?	Yes	Yes	Yes	Yes	Yes	Yes
District fixed effects?	Yes	Yes	Yes	Yes	Yes	Yes
Observations	1,159	2,390	873	791	802	831
R-squared	0.179	0.117	0.221	0.157	0.175	0.178
Wald tests (p-values):						
β(*Treatment*) > β(*Control*)	0.72	0.99	0.95	0.39	0.17	0.64

Notes: These are linear probability model estimates, with standard errors are in parentheses, clustered at village level. ***p < 0.01, **p < 0.05, *p < 0.10. “All controls” includes controls for ownership of other livestock and total TLUs (livestock wealth), as well as the full set of socioeconomic controls described in Table, a gender dummy and monthly dummies for child age, as well as district fixed effects.

An alternative to modelling a dichotomous indicator of whether the household produced any milk is to specify the household’s estimate of the quantity of milk it produced in the past 12 months, which we measure as the log of litres per child. OLS coefficients estimates for this indicator are reported in Table 6. These coefficients are significant in the 6–23 month and 12–23 month brackets, and marginally insignificant in the 6–17 month bracket. In the 6–23 month range the coefficient implies that increasing milk production by 10% would reduce stunting by 0.08 percentage points. The coefficients are imprecisely estimated, however, and likely suffer from attenuation bias related to the significant challenges that respondents have in accurately answering 12-month recall questions. Overall, though, the results are broadly consistent with the results from Table 4.

**Table 6:** OLS and IV estimates of the association between HAZ and the log of milk production per child.

	(1)	(2)	(3)	(4)	(5)	(6)
	6-23 months	24-59 months	6-17 months	12-23 months	18-29 months	24-35 Months
Log quantity of milk produced	0.084**	0.008	0.083	0.080*	0.031	−0.017
	(0.034)	(0.024)	(0.050)	(0.040)	(0.037)	(0.043)
All controls?	Yes	Yes	Yes	Yes	Yes	Yes
District fixed effects?	Yes	Yes	Yes	Yes	Yes	Yes
Observations	1,159	2,390	873	791	802	831
R-squared	0.192	0.124	0.201	0.179	0.151	0.158

Notes: These are least squares estimates, with standard errors are in parentheses, clustered at village level. ***p < 0.01, **p < 0.05, *p < 0.10. All regressions control for ownership of other livestock and total TLUs (livestock wealth), as well as the full set of socioeconomic controls described in Table, a gender dummy and monthly dummies for child age, as well as district fixed effects.

## 4 Exploring the relationship between dairy production and breastfeeding

**Fig. 4:**
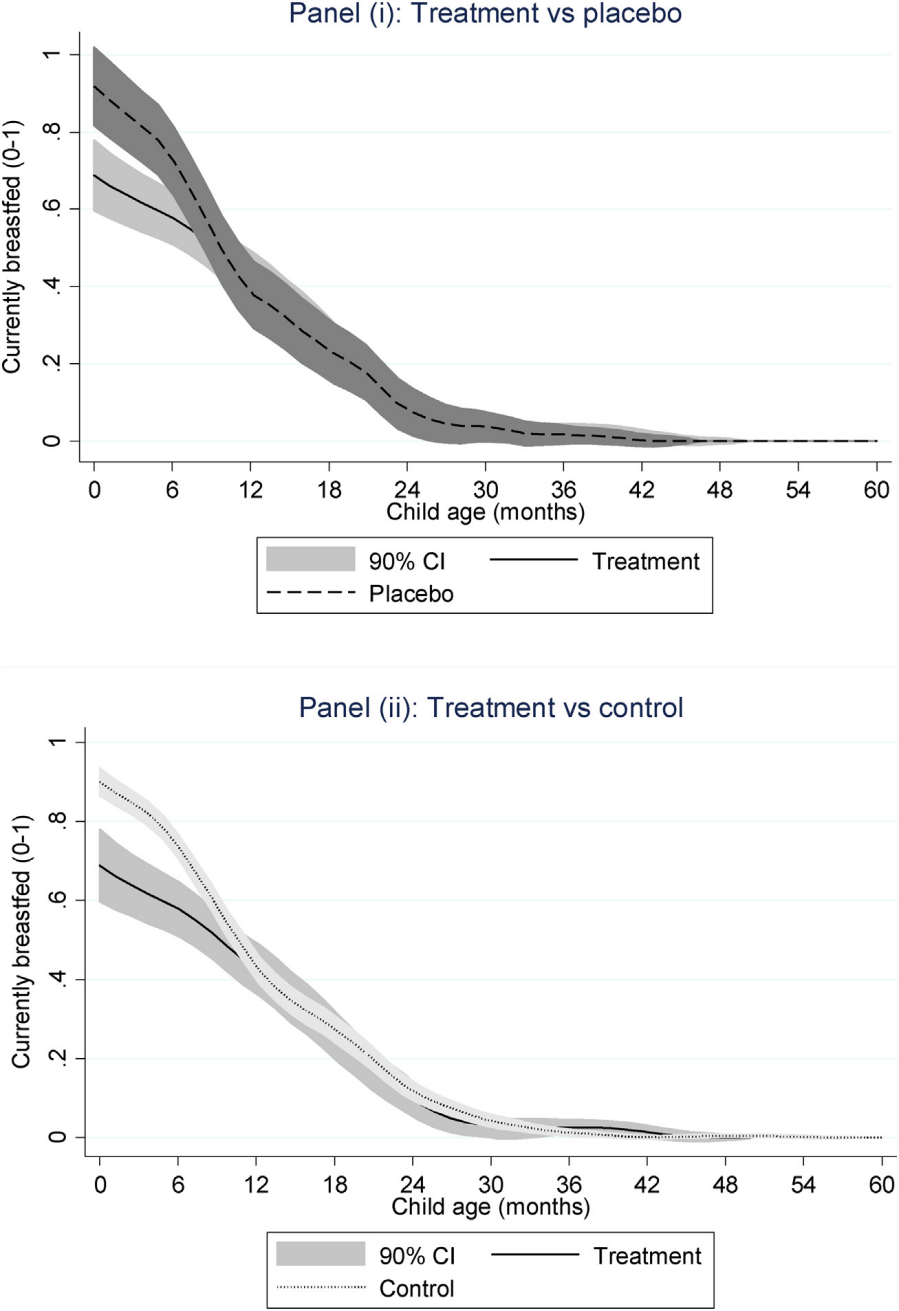
A local polynomial smoothing graph of breastfeeding status by child age for households that have and have not produced dairy.

One concern with the results reported in Fig. 2 is that many children in the *treatment* group consume dairy at young ages (Fig. 3) when it may be harmful to the infant digestive system (FAO, 2013), or may substitute for breastmilk, which has been linked with a range of desirable health outcomes (PAHO/WHO, 2003). In this section we explore whether there might be substitution between breastmilk and household supplies of dairy milk. Fig. 4 plots breastfeeding status by child age with comparisons between *treatment* and *placebo* (Panel i) and *treatment* and *control* (Panel ii). The results show that, from birth to around 8 months of age, dairy-producing households are significantly less likely to breastfeed their children. Above this age range there is no significant difference in breastfeeding rates. This suggests that access to dairy milk may have a negative spillover on breastfeeding practices in the critically important 0–5 months age range when it is strongly recommended for infants to be exclusively breastfed.

In Table 7 we estimate a linear probability model with current breastfeeding status as the dependent variable, with the usual battery of control variables included to see whether the results in Fig. 4 are robust to a multivariate model. This is indeed the case: children 0–11 months from *treatment* households are around 21.7% less likely to be breastfed than children from *control* households, and there is a similar statistically significant difference between *treatment* and *placebo*. The evidence therefore suggests that easy access to dairy milk greatly reduces the incentive for mothers to breastfeed.

**Table 7:** Linear probability model estimates of the association between current breastfeeding status cow ownership among children 0–11 months of age.

	(1)
Dependent variable:	Currently breastfed
*Treatment group (vs control)*	−0.217**
	(0.087)
*Placebo group (vs control)*	−0.033
	(0.068)
All controls?	Yes
District fixed effects?	Yes
Observations	759
R-squared	0.321
Wald tests (p-values):	
β(*Treatment*) > β(*Control*)	0.015**

Notes: These are linear probability estimates, with standard errors are in parentheses, clustered at village level. ***p < 0.01, **p < 0.05, *p < 0.1.

## 5 Conclusions

Despite strong biological evidence on the links between dairy consumption and child growth, and substantial empirical evidence from developed country populations, surprisingly little research has documented the impacts of regular consumption of dairy products on child growth in developing countries. Recent economic research has instead examined associations between cattle ownership and child growth, but only looked at East African populations. And to our knowledge none of this research has examined substitution of dairy milk for breast milk. In this paper we examined these associations in Bangladesh where we were able to distinguish between cows that produced milk in the past 12 months and those that did not. This dichotomy served two purposes. First, by focusing more specifically on herds that have actually produced milk our estimates may more closely approximate the growth benefit of the latent variable of interest, the regular consumption of dairy products. Second, “treating” children with cows that have not produced milk offers a potentially meaningful placebo test.

We find results broadly consistent with the findings of East African settings. Similar to Hoddinott et al. (2015), we were able to disaggregate results by age and show that the benefits of cattle ownership (or regular supply of dairy products) emerges primarily in the 6–23 month critical window of child growth. However, Hoddinott et al. (2015) find an estimated impact of owning at least one cow of 0.21 standard deviations, without knowing whether the cow produced milk or not. When we replicate that approach we find an impact of 0.35 standard deviations for owning any cow (results available on request), whereas the results reported above suggest an estimated impact of 0.52 SD for owning at least one cow that produced milk in the past 12 months. Hence the associations estimated in this paper are substantially larger and partially pertain to the use of a better proxy for regular milk consumption. Our point estimates are very similar in magnitude to those of Rawlins et al. (2014) from Rwanda, and Kabunga et al. (2017) from Uganda, even though both of those studies focus on improved (high-yielding) cattle varieties rather than ownership of any type of dairy cow. This literature therefore corroborates existing evidence on the importance of cow’s milk for linear growth, which mostly stems from more developed settings (Iannotti et al., 2013; de Beer, 2012; Hoppe et al., 2006).

Given that less than a quarter of rural Bangladeshi children consumed dairy products over the previous 24 h, and that almost half of rural Bangladeshi children are stunted, increasing dairy consumption among children and women of childbearing age should be a central priority for nutritional strategies in Bangladesh. The best means of doing so is unclear, however. With exceptionally high population densities even in rural areas, Bangladesh has no clear comparative advantage in large-scale dairy production and may ultimately need to rely more on milk powder imports, which are still heavily taxed with a tariff of 25%. Additional constraints may be more cultural in nature. Like many East Asian countries, Bangladesh has no strong tradition of milk consumption. However, several East Asian countries, such as Thailand and Vietnam, have been extremely successful in increasing dairy consumption through combinations of imports and rapid growth in domestic production, as well as marketing campaigns and school feeding programs aimed at increasing nutritional knowledge and consumer demand for dairy products (FAO, 2008). However, our results also provide a further rationale for utilizing campaigns aimed at improving nutritional knowledge; that there is a need to reduce the perceived substitutability between dairy products and breastmilk.

## Footnotes

1 Observational evidence on the linkages between dairy consumption and child growth is also limited by the paucity of high quality data on “usual diets” in developing countries. The widely used Demographic Health Surveys (DHS) now 24-hour recall indicators of food consumption, but this is likely a relatively poor indicator of regular consumption of dairy products in many settings. Some children who did consume milk in the last 24 hours may not be regular consumers of milk, and vice versa. This misclassification may lead to attenuation biases when trying to estimate the impacts of milk consumption on child growth with observational data.

2 In addition to these studies, other studies look at livestock ownership and HAZ without specifically distinguishing livestock breeds nor dairy cow ownership specifically. Mosites et al. (2015) find significant negative associations between total livestock ownership and stunting in Ethiopia and Uganda, but not in Kenya. Azzarri et al. (2015) apply an instrumental variable (IV) approach to a smaller Ugandan household survey and find no impact of large ruminants on stunting outcomes, but some negative associations with underweight status.

3 Here we only review published studies on cattle ownership and child growth. Rawlins et al. (2014) review several much earlier unpublished studies on this topic, though all involve very small sample sizes, and all still pertain to East Africa (Kenya, Malawi, Rwanda).

4 Other potentially endogenous determinants of the lactation cycle include seasonal diseases and heat stress, land access, poor management practices related to oestrus detection, poor animal nutrition, and poor access to male cattle or artificial insemination services (Shamsuddin et al., 2007; Kamal, 2010). Kamal (2010) writes:

5 Applying household fixed effects to look at within-household differences in siblings’ exposure to dairy production would be possible in principle, but would require an even larger sample than we use in this paper. This is because we already split the sample into 12-month age brackets, only 25% of households own dairy cows, and only a quarter of these have not produced milk in the past 12 months. Fertility rates in Bangladesh are now sufficiently low (2.9) that relative few households have multiple children in the 0-59 month age bracket, let alone the 6-24 month window of interest.

6 Indeed, BIHS data on milk sourced from own consumption shows that milk-producing households acquire around three-quarters of their household milk supply from own production, suggesting they still rely substantially on markets to supplement household consumption requirements.
